# Prevalence of post traumatic stress disorder in children with mild traumatic brain injury

**DOI:** 10.1192/j.eurpsy.2021.1205

**Published:** 2021-08-13

**Authors:** P. Kallianezos, M. Bakola, K.S. Kitsou, C. Petropoulos, X. Sinopidis, P. Gourzis, E. Jelastopulu

**Affiliations:** 1 Pediatric Surgery, General Children’s Hospital of Patras, Patras, Greece; 2 Postgraduate Program Of Public Health, Medical School, University of Patras, Patras, Greece; 3 Mathematics Department, Division Of Statistics, Probability And Operational Research, Univestity of Patras, Patras, Greece; 4 Pediatric Surgery Clinic, University Hospital of Patras, Patras, Greece; 5 Department Of Psychiatry, School of Medicine, University of Patras, Patras, Greece; 6 Department Of Public Health, Medical School, University of Patras, Patras, Greece

**Keywords:** PTSD, Children, brain, injury

## Abstract

**Introduction:**

Children with traumatic brain injury (TBI) are at risk for post-traumatic stress disorder (PTSD). The vast majority of TBI are of mild severity (MTBI), however, they may develop persistent neurophysiological symptoms.

**Objectives:**

The purpose of this study was to investigate the incidence of PTSD in children with MTBI in Western Greece.

**Methods:**

A one-year prospective study was conducted at the Children Hospital of Patras. A total of 175 children aged 6-14 years screened for risk of PTSD at one-week and one-month post-injury, completing the Child Trauma Screening Questionnaire (CTSQ). The Children’s Revised Impact of Event Scale (CRIES 13) was administered to the parents, to inquire their assessment of PTSD in the children. Statistical analysis was performed with IBM SPSS v.22.0

**Results:**

There were 59 (33.7%) children (27.2% boys, 45.9% girls) whose screen result was at risk. At the rescreening one-month postinjury, 9.9% were still at risk. Parents assessed presence of PTSD in 19% of their children at one-week and in 3.9% at one-month post-injury. There was a positive correlation between parenting and child reporting on symptoms of PTSD in children. However, 23.4% mistakenly estimated their children did not experience stress while in fact they did and 24.2% mistakenly estimated the contrary.

**Conclusions:**

The findings revealed the risk of PTSD even in mild TBI, justifying thus the screening to identify these children for intervention strategies. On the other hand, the rescreening demonstrated that not all at-risk children required intervention, since a natural remission in PTSD symptoms was observed one-month post-injury.

